# A Contraband Detection Scheme in X-ray Security Images Based on Improved YOLOv8s Network Model

**DOI:** 10.3390/s24041158

**Published:** 2024-02-09

**Authors:** Qingji Gao, Haozhi Deng, Gaowei Zhang

**Affiliations:** 1 Robotics Institute, Civil Aviation University of China, Tianjin 300300, China; 2 School of Electronic Information and Automation, Civil Aviation University of China, Tianjin 300300, China

**Keywords:** contraband detection, YOLOv8s, attention mechanism, deformable convolution net, pigeon-inspired optimization

## Abstract

X-ray inspections of contraband are widely used to maintain public transportation safety and protect life and property when people travel. To improve detection accuracy and reduce the probability of missed and false detection, a contraband detection algorithm YOLOv8s-DCN-EMA-IPIO* based on YOLOv8s is proposed. Firstly, the super-resolution reconstruction method based on the SRGAN network enhances the original data set, which is more conducive to model training. Secondly, DCNv2 (deformable convolution net v2) is introduced in the backbone network and merged with the C2f layer to improve the ability of the feature extraction and robustness of the model. Then, an EMA (efficient multi-scale attention) mechanism is proposed to suppress the interference of complex background noise and occlusion overlap in the detection process. Finally, the IPIO (improved pigeon-inspired optimization), which is based on the cross-mutation strategy, is employed to maximize the convolutional neural network’s learning rate to derive the optimal group’s weight information and ultimately improve the model’s detection and recognition accuracy. The experimental results show that on the self-built data set, the mAP (mean average precision) of the improved model YOLOv8s-DCN-EMA-IPIO* is 73.43%, 3.98% higher than that of the original model YOLOv8s, and the FPS is 95, meeting the deployment requirements of both high precision and real-time.

## 1. Introduction

A security check is the first line of defense to guarantee people’s travel safety as an increasing number of people choose to travel via different methods of transportation due to the rapid development of public transportation and the transportation industry. Especially in the realm of civil aviation, passenger behavior gone wrong is mostly to blame for frequent aircraft mishaps. Airports have implemented very stringent security protocols to optimize the personal safety of travelers. X-ray security is currently one of the most popular security technology methods [1]. Airports, high-speed rail stations, and other public areas utilize X-ray security equipment due to its low cost, high recognition, and non-destructive testing advantages. Trained manual inspectors visually analyze X-ray scanning photos to make sure there is no danger [2,3]. Sometimes, it takes only a few seconds during peak hours to assess whether a piece of luggage contains harmful goods [4]. Due to the sheer volume of luggage that security inspectors must check and the ease with which contraband can be obscured by other objects, manual identification can be challenging. As a result, the inspector’s identification experience and weariness can occasionally affect the detection results, which can result in missed or false positives [5,6]. To guarantee the security of public transportation, it is crucial to create an intelligent, effective, and precise detection and recognition algorithm.

Traditional target detection algorithms have made many contributions to the classification and recognition of security contraband. At present, a commonly used method is based on the image features of a package, and the contour detection algorithm is used to extract the package. The typical features of the package image are the gray feature and the edge feature. Wu Ying et al. extracted the image’s golden zone using edge detection operators [7]. And, Mei Hongyang et al. extracted the outlines of moving objects using edge features [8]. Su Bingshan et al. proposed a new detection and classification method. Contourlet transform was used to decompose the images scanned using X-rays, and the decomposed co-occurrence matrix, Tamura texture feature and histogram feature were extracted [9]. Finally, the feature vectors of these three features were linked in series to obtain the joint feature vector. Wang Yu et al. proposed a study on the classification of foreign objects in X-ray images based on computer vision, which mainly uses Tamura texture features and random forest to automatically identify and classify prohibited objects in X-rays [10]. Han Ping et al. proposed a method of adaptive sinusoidal gray transform to achieve two-level enhancement, which can significantly improve image quality [11]. The ability of feature extraction and capture of traditional methods is poor, and its robustness to the diversity of targets is low. As a result, the detection model lacks a particular generalization ability and cannot be well applied to a large amount of data.

The convolutional neural network (CNN) has been widely used in image processing and analysis in recent years, thanks to the high-speed development of deep learning methods [12], and many computer vision tasks, such as face recognition, behavior recognition, medical image processing, and automatic driving, have made significant progress, reaching the most advanced and efficient detection performance [13,14]. At the same time, researchers are also looking at the field of X-ray security contraband detection. Before that, the main method for image recognition and classification was the bag-of-visual-words (BoVW) model, which extracted visible words through feature descriptors [15]. The k-means algorithm was used to cluster features and combine visible words with similar meanings [16]. It is also possible to create vocabularies for classification via RF, SVM, or sparse representation [17,18]. The application of the standard BoVW method to the classification and detection of X-ray images can improve the performance. Mery et al. compared the effectiveness of X-ray contraband detection techniques based on deep learning, sparse representation, the visual bag-of-words model, and traditional pattern recognition schemes [19,20,21]. They discovered that the deep learning approach produced the best results [2]. Wu Haibin et al. added improved void convolution based on the YOLOv4 algorithm, used a multi-scale aggregation of context information, and finally optimized the candidate boxes via the k-means clustering algorithm, which eventually improved the detection accuracy [22]. Dong Yishan et al. proposed an improved YOLOv5 X-ray contraband detection model. Based on the YOLOv5 algorithm, the model introduced an attention mechanism, border fusion and data enhancement strategies to improve detection accuracy [23]. Because the volume of contraband is smaller than that of luggage, and the ordinary network model is weak in detecting small targets, it is easy to cause the problem of missing and misdetection. To solve the above problems, Zhang Youkang et al. added three detection modules based on the one-stage target detection network SSD framework [24] and proposed a multi-scale contraband detection network ACMNet suitable for X-ray security inspection images, and they achieved satisfactory results [25]. Based on the improvement of the YOLOv3 network model, Guo Shouxiang et al. changed its backbone network to a new backbone network composed of two darknets and introduced a feature enhancement module to improve the detection effect of small targets [26]. To detect small residual items on X-ray clothing images, Rong Gao et al. proposed combining the feature pyramid network (FPN) with the Faster R-CNN algorithm [27,28,29]. The combination of FPN and Faster R-CNN can make better use of feature maps with higher resolution. Wei et al. proposed an attentional module DOAM for removing occluding baggage. When faced with a high degree of occluding baggage, DOAM can bring a good improvement effect [30]. Zhang Na et al. proposed a dangerous-goods-detection algorithm for X-ray security based on the improved Cascade RCNN network to improve the detection accuracy by enhancing local feature learning and changing the weight proportional coefficient [31]. Aiming at solving the problems of false detection and missed detection of contraband in X-ray security screening scenarios, You xi et al. proposed an adaptive security contraband detection method XPIC R-CNN based on Cascade R-CNN that integrates spatial attention, which significantly improves the average detection accuracy, but a large amount of computation leads to low real-time performance [32]. The aforementioned techniques have made significant strides toward improving the accuracy of security contraband detection based on deep learning; yet, the following issues persist: (1) In the case of severe overlap occlusion, it is easy to confuse the object to be inspected with the background, and similar feedback will be obtained when images are transferred to the network to extract features through the convolutional layer, thus reducing the recognition and detection accuracy of contraband. (2) Due to the fixed sampling position, the ordinary convolution cannot adapt to the actual detection target receptive field, which affects the ability of the model to extract features and ultimately results in missing detection. The size and shape of various contraband items vary greatly. (3) The setting of the anchor frame depends on manual marker setting, resulting in a weak generalization ability of the initial value of the anchor frame and low detection accuracy for small samples and small targets, which affects the final detection accuracy of the model.

Since the single-stage object detection model YOLO was published, it has been widely considered and applied in the industry. In recent years, the YOLO series algorithm has been updated and iterated in several versions. The Ultralytics team proposed the YOLOv8 version in 2023, which not only meets the requirements of real-time performance, but also has a lighter network structure and eliminates the anchor frame mechanism while meeting high detection accuracy. Currently, a variety of real-world issues have been resolved by the YOLOv8 model and its enhanced approach such as dense pedestrian detection [33,34], road damage detection [35], gesture recognition [36], etc., but there are few examples of dangerous goods detection. Therefore, in view of the above three problems, this paper takes the YOLOv8s model as the base framework and makes improvements from four aspects: data enhancement, fusion deformable convolution (DCNv2), added attention mechanism (EMA), hyperparameter optimization, and finally, the model’s detection accuracy used for contraband detection. The main innovations are as follows:

(1) The data enhancement of the X-ray security inspection data set and the introduction of SRGAN super-resolution reconstruction technology can improve the resolution and brightness of the original image, make the appearance and shape of the items to be inspected in the picture more precise and have more information, which is conducive to the feature extraction operation of the model.
(2) To enhance the feature extraction network and improve the ability of the model to detect contraband on different scales, DCNv2 (deformable convolution net v2) is introduced into the backbone network. An EMA (efficient multi-scale attention) module is proposed to implement the adaptive calibration of feature map channels, thereby improving the model’s attention to the target region and improving the reduction in complicated background interference and the overlapping occlusion issue.
(3) A pigeon colony algorithm based on a cross-mutation approach is developed to improve the learning rate parameters in the model’s hyperparameters by mimicking the behavioral traits of flock homing. The algorithm’s features include a broad search range and quick response time. The model’s initial learning rate is generated by the improved pigeon-inspired optimization, and the mAP index serves as the fitness function. Continuous iteration is applied to obtain the optimal learning rate, which is then translated into a corresponding mAP value that improves target detection accuracy.

The remaining contents of this paper are as follows: Chapter 2 introduces the principle of YOLOv8s and the related improved model mentioned above; Chapter 3 mainly presents the test experiment and results from the analysis; and Chapter 4 concludes the full text.

## 2. Materials and Methods

### 2.1. Data Enhancement 

Generally speaking, in deep learning, the higher the resolution and the greater the number of image datasets, the better the performance of the model trained using these datasets. When the number of data sets is insufficient, the recognition accuracy of the model will be low, the recognition ability will be weak, and the generalization ability will be inadequate. In actuality though, gathering high-quality scene image data is frequently challenging because of the environment’s complexity and unpredictability as well as the capacity limitations of the acquisition equipment. As a result, picture augmentation technology is required to prevent it from overfitting and increase the model’s recognition accuracy.

**Figure 1:**
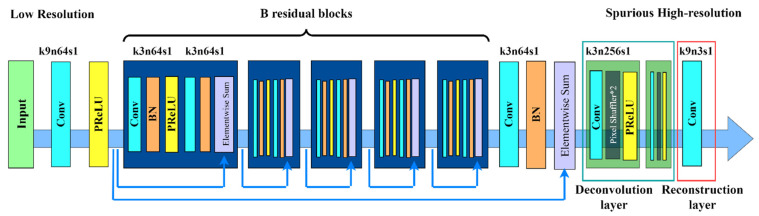
The network structure of the SRGAN generator.

**Figure 2:**
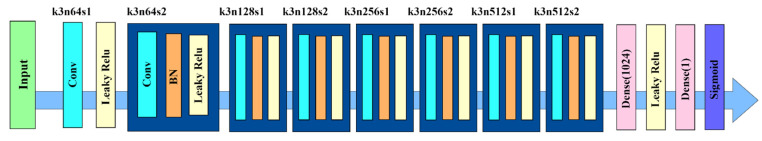
The network structure of the SRGAN discriminator.

The super-resolution reconstruction technique is applied in this research to improve the images. Increasing and enhancing an image is referred to as super-resolution, whereas super-resolution reconstruction technology is the technique of reconstructing a high-resolution image from one or more low-resolution photographs. Its purpose is to recover image details, improve clarity, and improve visual quality. Traditional reconstruction approaches are classified into two categories: interpolation-based methods and regularization-based methods. However, the results obtained in this manner are unsatisfactory, resulting in blurred images. As a result, super-resolution reconstruction technology based on deep learning has been developed and has proven to be a potent and effective answer to the growth of artificial intelligence. It can generate incredibly detailed high-resolution photos, which significantly improves the reconstruction effect. Because the resolution of the picture data set scanned by the security detector in this article is low, it is important to convert the image to HD using the SRGAN network, a super-resolution reconstruction approach based on deep learning. SRGAN is a hybrid of the generative adversarial network (GAN) and the deep convolutional neural network (CNN) [37,38]. The SRGAN network with a generative adversarial network model can obtain more accurate amplification results than traditional super-resolution reconstruction techniques, resulting in natural images with excellent perceptual quality. Figure 1 and Figure 2 represent the structure of the generator network and the discriminator network, respectively.

The generator network and the discriminator network make up the two fundamental components of the SRGAN method. An input image with poor resolution is first sent to a 9 × 9 convolution layer by the generator network design. Next, it is received as input by the PReLU function. These values are then entered into residual blocks, each containing two 3-by-3-sized 64-pixel convolution layers, using the ReLU function as the activation function after the residual block [39]. Lastly, the super-resolution image is formed as the output after two deconvolution layers are employed for upsampling to increase the image size. Eight convolutional layers make up the discriminator network structure, which is in charge of differentiating between the created super-resolution image and the actual high-resolution image. First, the convolution layer is used for the input image so that it extracts features from the input image and passes them to the Leaky ReLU activation function. It is then processed by seven modules, which include a convolution layer, a batch normalizing layer, and the Leaky ReLU function. Finally, the authentication results are produced by the fully connected layer and the Sigmoid function.

**Figure 3:**
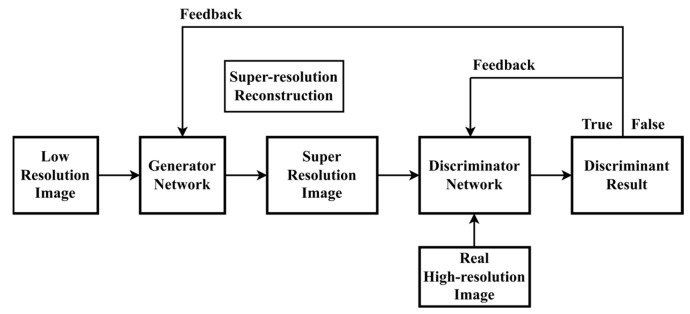
The super-resolution reconstruction training procedure.

In contrast to a single-structure deep learning network, a generative adversarial network generates objects using a generator network and a discriminator network for adversarial learning. Both generators and discriminators learn by competing with one another. The generator’s objective is to convert a low-resolution (LR) image into a super-resolution (SR) image. The discriminator aims to discriminate between true high-resolution images (HR) and super-resolution images (SR) and to provide the generator and discriminator models with the discriminant results. The generator and the discriminator both update the relevant parameters at the same time; the generator continues to fool the discriminator by generating a realistic super-resolution image that captures the image’s details and overall visual features, and the discriminator provides feedback to the generator on developing the image to encourage the generator to learn more efficiently and minimize losses. In this paper, the DIV data set is utilized for 300 rounds of training to achieve the appropriate weight, and then it is applied for super-resolution reconstruction of the security detector data set to produce high-resolution images. Figure 3 depicts the training procedure for super-resolution reconstruction.

### 2.2. The Design of the YOLOv8s-DCN-EMA Model to Optimize the Detection Accuracy of Contraband

#### 2.2.1. The YOLOv8s Network Structure

In the division of security contraband, it is necessary to identify and detect dangerous goods. Based on the location of the detected contraband, the size of the item is estimated to determine whether the item belongs to the category of contestable goods. 

**Figure 4:**
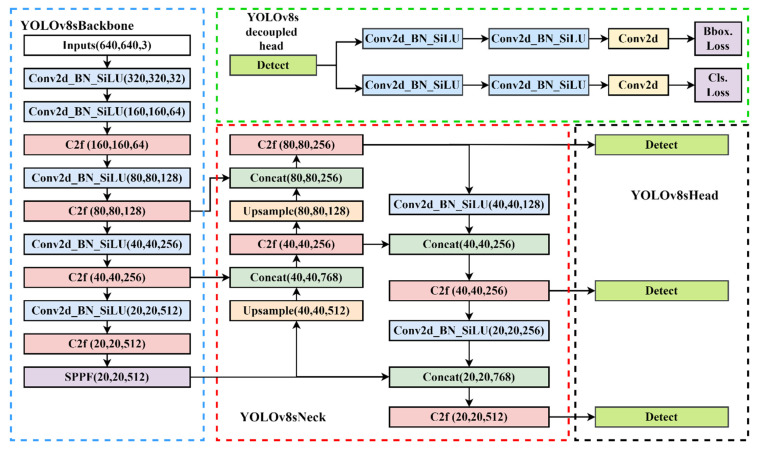
The network structure of YOLOv8s model.

The YOLO series detection algorithm, which is one of the most well-known object detection algorithms, divides the image into multiple networks, predicts the bounding box within each grid and the category of objects it contains, and uses the non-maximum suppression (NMS) algorithm to eliminate overlapping bounding boxes, which has the characteristics of fast speed and high precision. YOLOv1 has a relatively quick detection speed, but its detection effect is not optimal for objects that are relatively close together and small targets [40]. The YOLOv2 algorithm employs the Darknet19 network, which is very adaptable and can accommodate images of varying sizes [41]. To enhance its capacity to detect data at various scales, YOLOv3 incorporates the spatial pool pyramid and feature pyramid modules [42]. YOLOv4 introduces mish activation functions to improve accuracy [43]. YOLOv5 introduces SPPF and C3 modules to optimize the detection performance. To achieve the goal of continuously enhancing the network learning ability without destroying the original gradient path, YOLOv7 introduces the E-ELAN module, which uses extension, shuffle, and merge cardinality [44]. This improves the ability of feature extraction and semantic information expression. The YOLOv8 detection algorithm used in this paper, proposed by Glenn Jocher, is improved on the YOLOv5 algorithm. Compared with previous generations of networks, YOLOv8 further optimizes the network structure and improves the comprehensive performance of object detection. The network structure of the YOLOv8s model is shown in Figure 4.

The YOLOv8 model can be categorized into YOLOv8n, YOLOv8s, YOLOv8m, YOLOv8l, and YOLOv8x from small to large based on the parameters of the model. The YOLOv8s model is chosen as the detecting head in consideration of the size of the goods and the precision and real-time detection of contraband. The input layer, the neck layer of the feature enhancement module, the head layer of the output end, and the backbone layer of the network backbone module make up the four main components of the YOLOv8s network topology, which is depicted in the picture below.

**Input**

Since YOLOv4, Mosaic, Mixup and other image enhancement technologies have been added to the data preprocessing module. YOLOv8 mainly adopts the processing strategy of YOLOv5. It includes four augmentation methods: Mosaic, Mixup, random perspective and the HSV augment. Among them, Mosaic technology is used to enhance the data set. The Mosaic technique is used to randomly arrange and crop the four feature maps to enrich the background of the feature maps. Although image aliasing enhancement has many advantages, experience has shown it to reduce training effectiveness if used during the training process. Therefore, the YOLOv8 model will choose to turn off the Mosaic enhancement operation in the last 10 epochs during the training process, which can improve accuracy.

**Backbone**

**Figure 5:**
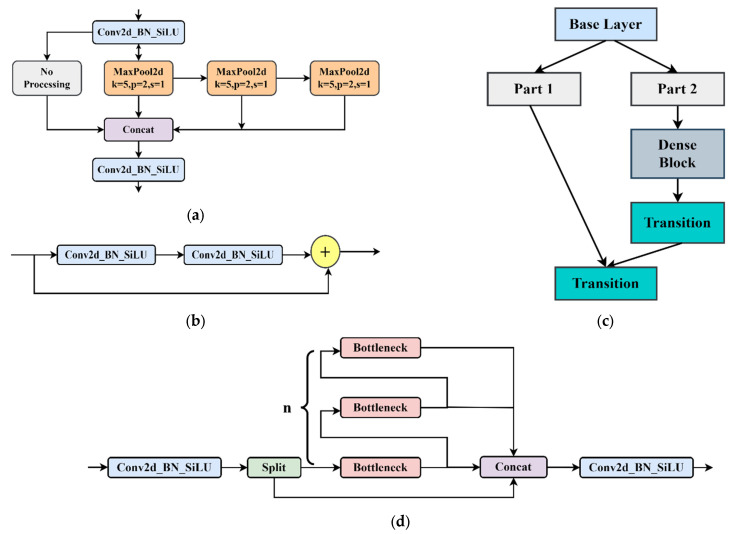
The schematic diagram of SPPF module, bottleneck module, CspLayer module, and C2f module. (**a**) SPPF module; (**b**) bottleneck module; (**c**) CspLayer module; (**d**) C2f module.

Backbone is the feature extraction part of the YOLOv8 model and uses the same idea of CSPNet. CSPNet is generally combined with ResNet, DenseNet, and other networks to optimize the network structure and reduce computing and memory consumption. Take DenseNet as an example. Through CSPNet, the underlying feature map is divided into two parts. One part is output via the original DenseNet and other modules, and the other part is directly combined with the output just mentioned. The backbone network comprises three modules: CBS, C2f, and SPPF. The CBS module is composed of a Conv2d convolution module, batch normalization and the SiLU function. The neck network refers to the ELAN structure design idea of YOLOv7, replaces the C3 module with the C2f structure, and optimizes the module structure, a bottleneck module connected by gradient flow. Furthermore, the network structure is lightweight, and rich gradient flow information can be obtained. Therefore, the C2f structure can enhance the feature fusion ability of the convolutional neural network and improve the reasoning speed. The SPPF is optimized on the structure of the SPP module. The SPPF module changes the original 3 convolution cores of different sizes into 3 5 × 5 convolution cores. This is because two 5 × 5 convolution nuclei in a series have the same effect as one 9 × 9 convolution kernel. Similarly, three 5 × 5 convolution nuclei in a series are equivalent to one 13 × 13 convolution kernel. Multiple convolution nuclei in a series can reduce the computation of the network and improve the detection efficiency. Figure 5 illustrates some typical modules in the YOLOv8s network model.

**Neck and Head**

YOLOv8 continues the FPN + PAN idea from YOLOv5 but also removes the 11 convolutional layers before upsampling and directly upsamples the input features of different stages in the backbone feature extraction network, which can optimize the network structure and improve detection efficiency. In comparison to YOLOv5, the head section has undergone significant changes. The decoupling head structure replaces the original coupling detection head structure. According to the research, employing the current decoupling detection head for the same target detection task can expedite convergence and enhance detection accuracy. YOLOv8 also changes the previous anchor-base method and uses the idea of the anchor-free method to no longer predict the offset of the anchor frame, thus improving many problems caused by the anchor frame.

#### 2.2.2. The EMA Attention Mechanism

The initial YOLOv8s model’s C2f layer had an EMA attention mechanism integrated with it to handle the issue of missing and falsely detecting various types of contraband in the intricate airport security scene.

EMA splits an input feature map into G sub-features in the channel dimension direction to learn distinct meanings. EMA extracts the attention weight descriptors of the grouping feature maps using three different methods at the network level because the large local receptive fields of neurons are capable of gathering multi-scale spatial information. The 1 × 1 branch contains two parallel routes, whereas the 3 × 3 branch contains the third route. The authors use two 1D global averaging pooling operations in the 1 × 1 branch to encode the channels in both spatial directions, respectively, and only one 3 × 3 kernel is stacked in the 3 × 3 branch to capture the multi-scale feature representation. This reduces the computational effort and captures the dependencies between all channels [45].

Cross-space learning has been studied and used in many computer vision problems since it can currently build interdependence across spatial locations and channels. With EMA, you can attain a richer feature aggregation impact using a cross-spatial information aggregation approach with diverse spatial dimension orientations. To do this, the authors generate two tensors: one from a 1 × 1 branch and the other from a 3 × 3 branch. Following the 2D global average pooling operation to encode the branch output with the global spatial information, the output of the smallest branch is directly transformed into the matching dimensional shape before the channel feature joint activation process. The 2D global averaging pooling operation formula in this case is as follows:

(1) $$z_{c}=\frac{1}{H\times W}\sum_{j}^{H}\sum_{i}^{W}x_{c}(i,j)$$

*C*, *H*, and *W* represent the feature map’s number of channels, height, and width, respectively. 

**Figure 6:**
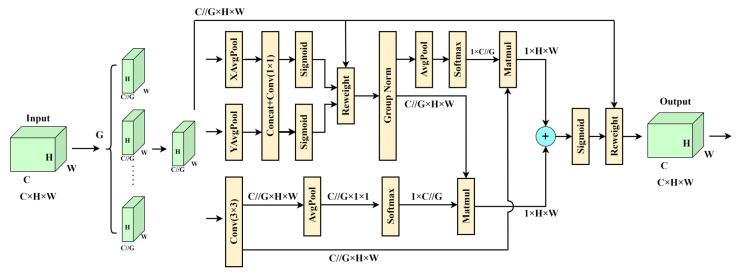
The structure diagram of the EMA attention mechanism.

Furthermore, global spatial information is also encoded in the branches utilizing 2D global average pooling procedures, and the branches are converted into appropriate dimensional forms just before the joint activation mechanism of the channel features. We then derive the second spatial attention diagram, which preserves the exact spatial position data. Lastly, two created spatial attention weight values are aggregated to create the output feature maps within each group. The structure of the EMA attention mechanism is shown in Figure 6.

In this paper, the EMA attention module is added to the neck end of YOLOv8s for the following reasons: (1) The EMA attention module is relatively computationally small, has lower model complexity, and has higher computational efficiency. (2) EMA not only outperforms other attention mechanisms such as SA, CBAM, CA, and ECA in terms of results, but it is also more efficient in terms of the required parameters [46,47,48,49]. (3) Good task adaptability: the EMA attention module is suitable for a wide range of visual tasks. The neck end of the YOLOv8 network is crucial for linking the prediction output head to the backbone network. Due to the particular structure of the neck end from bottom to top, features of different scales are fully integrated here, laying a foundation for future prediction, so the network structure of the neck end can greatly affect the performance of the algorithm. The following figure shows the network structure after the EMA module is added. This module is added after the Upsample layer in the up-sampling phase of PAN-FPN and after each C2f module in the down-sampling phase, before the convolution of the CBS module. The feature attention is strengthened before the feature fusion, so that the model can pay more attention to the smaller target, provide information on security inspection contraband, improve its identification and classification ability of dangerous goods and improve its positioning accuracy.

#### 2.2.3. The Design of Deformable Convolution Net v2 Module

In the actual X-ray security inspection images, the scale of contraband usually changes significantly, showing different shapes and sizes. Traditional convolution generally adopts a rectangular structure with fixed size and proportion to extract features from a certain position of the feature map, which makes it difficult for the receptive field to perceive the geometric deformation of the target, resulting in less effective information that can be extracted and easy-to-ignore critical feature information of the target. This work introduces DCNv2 with an adaptive geometric deformation mechanism to improve image feature extraction and improve the model’s capacity to learn the invariance of complex objects, thereby mitigating the aforementioned issues.

A further development of deformable convolution DCNv1 is deformable convolution DCNv2 [50,51]. To provide random sampling close to the present position, the deformable convolution DCNv1 principle adds an offset to each sample point based on conventional convolution. Assuming that the convolutional kernel has a 3 × 3 size, it has nine sampling points. Each of these nine sampling points has an offset variable assigned to it. It allows the convolutional kernel’s size and position to be dynamically adjusted based on the target object, improving the network model’s detection performance for objects with irregular shapes and sizes.

For a traditional two-dimensional convolution, the output feature map at some sampling point is $u_{0}$ defined as follows:

(2) $$y(u_{0})=\sum_{u_{k}\in \;R}x(u_{0}+u_{k})\cdot w(u_{k})$$

In Formula (2), $R=\left\{\left(-1,-1),(-1,0),(-1,1),(0,-1),(0,0),(0,1),(1,-1),(1,0),(1,1\right)\right\}$ is the receptive field area; $w(u_{k})$ is the weight of the convolution kernel at the sampling position $u_{k}$; $x(u_{0}+u_{k})$ is the feature of the input feature map x at the location $u_{0}+u_{k}$; and $u_{k}$ is the location element of R, that is, all sampling locations in the receptive field.

In a deformable convolution, the output eigenvalues $y(u_{0})$ are defined as follows:

(3) $$y(u_{0})=\sum_{u_{k}\in \;\;R}x(u_{0}+u_{k}+\Delta u_{k})\cdot w(u_{k})$$

In Formula (3), $\Delta u_{k}$ represents the learnable offset that the standard convolution increases at the sampling point, generally a decimal number, and $u_{0}+u_{k}+\Delta u_{k}$ is also a decimal number, where $\left\{\Delta u_{k}|k=1,2,\ldots ,N\right\},N=|R|$. Therefore, the sampling position of pixels $x(u_{0}+u_{k}+\Delta u_{k})$ after the introduction of the offset is usually implemented via the bilinear interpolation method. The formula for bilinear interpolation is as follows:

(4) $$x(u)=\sum_{v}x(v)\cdot \Phi (v,u)$$

where $u=u_{0}+u_{k}+\Delta u_{k}$ represents any position in the region; v represents all integer space positions in the input feature map, namely the four integer points around $u_{0}+u_{k}+\Delta u_{k}$; x(v) is used to represent the values of points at all integer positions in the feature graph; and Φ(v,u) represents the bilinear interpolation kernel function, which is a two-dimensional kernel and can be divided into two one-dimensional nuclei, expressed by Formula (5):

(5) $$\Phi (v,u)=\varphi (v_{x},u_{x})\cdot \varphi (v_{y},u_{y})$$

And, $\varphi (v,u)=\max\left\{0,1-\left|v-u\right|\right\}$.

The offset field layer is added to the output feature map in the deformable convolution DCNv1 operation process after the input picture has been extracted using the traditional convolution check. This allows for the acquisition of the bias domain of the convolution kernel’s sampling points. Since it contains shifts in the two-dimensional plane x and y direction, the number of channels is 2N. The size of the bias field obtained is consistent with the input feature maps, and the bias matrix of the sampling points can be obtained from it; thus, the offset $\Delta u_{k}$ is obtained. Considering that DCNv1 will introduce a lot of irrelevant background information to disturb the model when fitting the detection target, this paper proposes DCNv2 based on DCNv1 to improve the model’s ability to focus on the target image region. Compared with DCNv1, the output characteristic values $y(u_{0})$ of DCNv2 are defined as Formula (6):

(6) $$y(u_{0})=\sum_{u_{k}\in \;R}x(u_{0}+u_{k}+\Delta u_{k})\cdot w(u_{k})\cdot \Delta m_{n}$$

**Figure 7:**
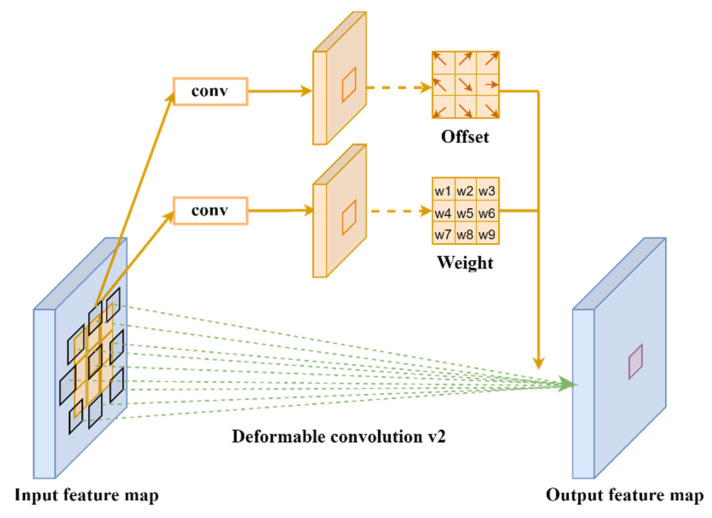
Illustration of 3 × 3 deformable convolution net v2.

The weight coefficient $\Delta m_{n}\;\;\;\;(0\leq \Delta m{\;}_{n}\;\;\leq 1)$ is introduced in the above formula. The amplitude of input features at different positions is adjusted by the modulation mechanism. The weight of each sampling point is learned so as to suppress irrelevant background information and reduce the interference of irrelevant factors. The process of DCNv2 is shown in Figure 7. 

In this paper, the deformable convolutional DCNv2 module is added to the backbone network of the YOLOv8s model. The backbone network plays a role in picture feature extraction. Blending and merging information on multiple levels can result in more extensive and accurate visual features of contraband. The second, third, and fourth C2f modules of the backbone network are replaced in this research by the deformable convolutional module C2f_DCN, which improves the model’s attention on small and medium-sized targets and lays the groundwork for future feature fusion. 

**Figure 8:**
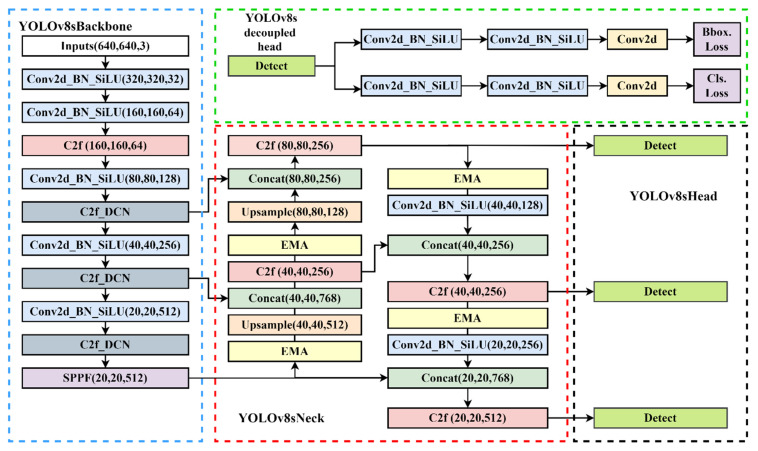
The network structure of the YOLOv8s-DCN-EMA model.

To sum up, the structure diagram of the YOLOv8s network with the EMA attention mechanism and deformable convolution added is shown in Figure 8.

### 2.3. The Pigeon-Inspired Optimization (PIO) Design Based on Cross-Mutation Operator to Optimize Learning Rate

#### 2.3.1. The Basic Theory of the PIO Algorithm

The PIO algorithm comprises two operators: the compass and map operator and the landmark operator [52]. The flock optimization model initializes the flock’s location and speed based on the compass operator, and the flock’s location and speed are updated during each iteration of the search process. In this case, speed and position are denoted as follows:

(7) $$X_{t}^{N_{t}}=X_{i}^{N_{t}-1}+V_{i}^{N_{t}}$$

(8) $$V_{i}^{N_{t}}=V_{i}^{N_{t}-1}\cdot e^{-R\times N_{t}}+\mathrm{rand}\cdot (X_{gbest}-X_{i}^{N_{t}-1})$$

In the above formula, R is the compass and map operator; rand is used to generate a random number in (0, 1); $X_{gbest}$ is the best position globally after the t−1 iteration loop; $V_{i}^{N_{t}-1}$ is the current velocity of the pigeon; and $X_{t}^{N_{t}}$ is the current position of the i-th pigeon in the Nt-th iteration.

The number of pigeons in each generation will be cut in half for the landmark operator. $N_{p}^{N_{t}}$ is used to represent the amount of pigeons in each generation, and $X_{center}^{N_{t}-1}$ is used to represent the center of the pigeons that are left. Therefore, the pigeons near their destination can use this as a landmark, as a reference direction of their flight.

(9) $$N_{p}^{N_{t}}=\frac{N_{p}^{N_{t}-1}}{2}$$

(10) $$X_{center}^{N_{t}-1}=\frac{\sum_{i=1}^{N^{N_{t}-1}}X_{i}^{N_{t}-1}\cdot fitness(X_{i}^{N_{t}-1})}{N_{p}^{N_{t}-1}\cdot \sum_{i=1}^{N^{N_{t}-1}}fitness(X_{i}^{N_{t}-1})}$$

where $N_{p}^{N_{t}}$ represents the number of the t generation pigeon flock. The fitness value is expressed as $fitness(X_{i}^{N_{t}-1})$, and the fitness value of each pigeon is evaluated and arranged to find the optimal path. Formulas (9) and (10) represent $N_{p}^{N_{t}}$ and $X_{center}^{N_{t}-1}$, respectively. Formula (11) is used to update the flock position:

(11) $$X_{i}^{N_{t}}=X_{i}^{N_{t}-1}+\mathrm{rand}\cdot (X_{center}^{N_{t}-1}-X_{i}^{N_{t}-1})$$

#### 2.3.2. Improved Strategy Based on Cross-Mutation Operator

For each individual in the population:

(12) $$X_{i}^{N_{t}-1}=(x_{1i}^{N_{t}-1},x_{2i}^{N_{t}-1},\ldots ,x_{ni}^{N_{t}-1})$$

The mutation operation is the core operation of the evolution operator, and the main purpose is to generate an intermediate individual through the mutation mechanism, whose mutation equation is as follows:

(13) $$W_{i}^{N_{t}}=\mu X_{r_{1}}^{N_{t}-1}+(1-\mu )\cdot F\cdot (X_{r_{2}}^{N_{t}-1}-X_{r_{3}}^{N_{t}-1})$$

(14) $$\omega =e^{1-\frac{N_{t\max}}{N_{t\max}+1-N_{t}}}$$

(15) $$F=F_{0}\times 2^{\omega }$$

(16) $$W_{i}^{N_{t}}=(w_{1i}^{N_{t}-1},w_{2i}^{N_{t}-1},\ldots ,w_{ni}^{N_{t}-1})$$

The i-th individual variation in the Nt-th generation produces an intermediate individual. $r_{1}$, $r_{2}$, $r_{3}$ are different from each other. They are taken from random numbers in $\left[1,N_{p}^{N_{t}}\right]$. F represents the constants between 0 and 1 and is determined jointly by $F_{0}$ and ω. Often called a variation factor or scaling factor, μ is a variation weight parameter used to expand the search capability of local and global scope.

To increase the diversity of interference parameter vectors, cross operation is introduced, and then the experimental variable becomes

(17) $$S_{i}^{N_{t}}=(s_{1i}^{N_{t}-1},s_{2i}^{N_{t}-1},\ldots ,s_{ni}^{N_{t}-1})$$

(18) $$s_{ji}^{N_{t}}=\left\{\begin{array}{l} w_{ji}^{N_{t}}\;\;\;\;\;\;\;\;\;\;\;rand(j)\leq CR\;\;\;or\;\;j=rnbi(i)\\ x_{ji}^{N_{t}-1}\;\;\;\;\;\;\;rand(j)>CR\;\;or\;\;\;j\neq rnbi(i) \end{array}\right.\;\;\;\;\;\;\;\;(i=1,2,\ldots ,N_{p}^{N_{t}}\;\;\;\;;\;\;\;\;j=1,2,\ldots ,n)$$

CR represents a crossover probability operator between 0 and 1, which determines the probability that the variable individual component value replaces the current individual score value; rand(j) is an arbitrary number in (0, 1); and rnbi(i) is an arbitrary integer belonging to 1 to n, ensuring that the candidate obtains at least one component value from the variation vector.

The classical method tends to converge too soon and settle into the local optimal solution because it lacks pigeon-to-pigeon contact. Meanwhile, there is a problem with inadequate diversity, and the algorithm’s pigeons’ location update formula has a poor global search capability. The adaptive differential mutation crossover operator, which is based on a previously improved strategy, is introduced. It can improve the algorithm’s ability to search locally and globally, increase population diversity, and change the position of pigeons by randomly selecting individuals within the flock.

The original compass operator, map operator and landmark operator will be changed in the algorithm of pigeon-inspired optimization improved by the mutation crossover operator.

Compass and map operators are

(19) $$X_{i}^{N_{t}}=X_{i}^{N_{t}-1}+V_{i}^{N_{t}}+W_{i}^{N_{t}}$$

The landmark operator is

(20) $$X_{i}^{N_{t}}=X_{i}^{N_{t}-1}+\mathrm{rand}\cdot (X_{center}^{N_{t}-1}-X_{i}^{N_{t}-1})+W_{i}^{N_{t}}$$

where $W_{i}^{N_{t}}$ is an improved adaptive mutation crossover operator.

#### 2.3.3. Improved PIO Algorithm 

According to the pigeon-inspired optimization mentioned in Section 2.3.1 and the improvement strategy mentioned in Section 2.3.2, an improved PIO algorithm, namely the IPIO (improved PIO) algorithm, is proposed, and the flow of the Algorithm 1 is as follows:
**Algorithm 1** Improved pigeon-inspired optimization **Step 1:** Set the flock parameters and initialize the flock, such as population number $\;N_{p}^{N_{t}}$, search dimension space D, compass operator R, map and compass operator’s maximum quantity of iterations $\;N_{t1}$, maximum amount of iterations for a landmark operator $\;N_{t2}$, maximum number of iterations $\;N_{t\max}$.**Step 2:** Determine the current ideal position by assigning each pigeon a random speed and position, then figuring out each one’s fitness value.**Step 3:** The population was crossed and mutated, and the pigeons’ positions were updated using the upgraded compass operator.**Step 4:** Compute the relevant fitness value, and then use the fitness value comparison to update the current global optimal location.**Step 5:** Verify if the compass operator’s maximum number of iterations has been reached. If yes, continue. Otherwise, go back to Step 3.**Step 6:** The population’s center location is calculated, then the population is mutated and crossed, and the enhanced landmark operator is utilized to update the pigeons’ location.**Step 7:** Calculate the corresponding fitness value, and then compare it to the current global ideal position.**Step 8:** Replenishing the population. **Step 9:** Check whether the maximum quantity of iterations of the landmark operator is reached. If yes, the global optimal solution is displayed. Otherwise, go back to Step 6.

**Figure 9:**
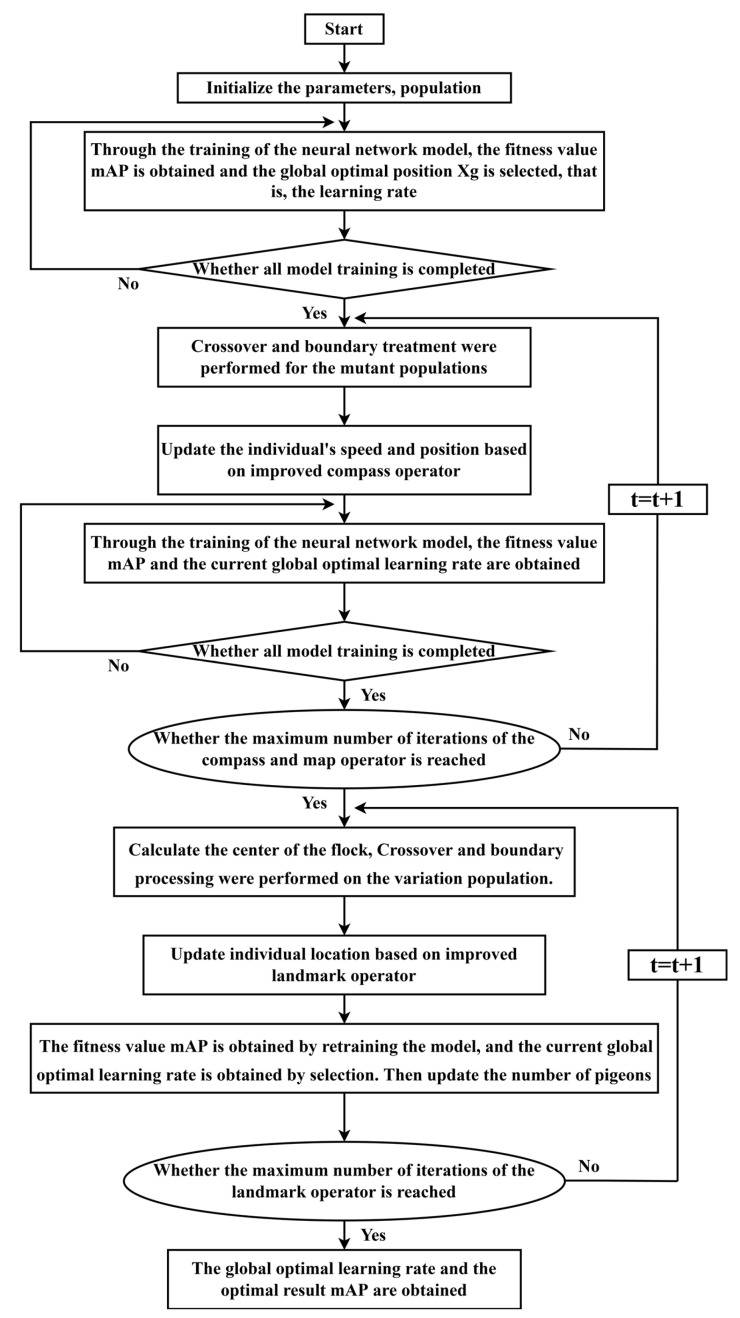
The flow chart of optimization of learning rate based on improved pigeon-inspired optimization algorithm.

This paper applies the improved pigeon-inspired optimization algorithm to the hyperparameter optimization of deep learning. The optimal learning rate and the optimal mAP (mean average precision) are found via an iterative search using the pigeon-inspired optimization algorithm based on the cross-mutation operator. The primary procedure is depicted in Figure 9:

## 3. Results and Discussion

### 3.1. YOLOv8s Detection Model Test Experiment Based on DCNv2 Deformable Convolution and EMA Attention Mechanism Optimization

#### 3.1.1. Dataset Construction and Experimental Environment Configuration 

X-ray scanning images were collected from airports, subways and other security systems as well as the internet. We selected, classified and labeled the collected pictures, and finally made 4800 data sets containing the contraband to be detected. The data set has eight common types of contraband, such as knives, scissors, and power banks. According to the construction principle of the training set, verification set and test set, the ratio of 7:1.5:1.5 is used to divide the data set. The model image has an input size of 640 × 640. Different detection and improved detection models were trained using the training set of security check baggage as comparative experiments, and the experimental parameters of other models were identical. Each image contained information such as the category and location of dangerous goods in the luggage. The operating system used in the experiment was 64-bit Ubuntu 20.04, the CPU was Intel(R) Core(TM) i7-11800H@2.30GHz, the GPU was NVIDIA GeForce RTX 3060, and the CUDA version was 11.6. The deep learning framework was the pytorch2.0 framework and the Python version was 3.9. For the setting of hyperparameters, the initial learning rate was set to 0.012, the number of training rounds was set to 100, and the batch was set to 8. The Mosaic enhancement was turned off for the last 10 rounds throughout the training process. The SGD optimizer was employed in the model to update the parameters iteratively. It can dynamically modify the learning rate to improve the convergence of the loss function.

#### 3.1.2. Experimental Evaluation Indexes

To judge the effect of the model, some evaluation indexes are needed, which usually contain four factors: true positive (TP), true negative (TN), false positive (FP), and false negative (FN). 

In object detection, the algorithm is measured by the following indicators:

(1) Precision

(21) $$Precision=\frac{TP}{TP+FP}$$

(2) Recall rate

(22) $$Recall=\frac{TP}{TP+FN}$$

(3) Balanced score (F1 Score)

(23) $$F1\;Score=2\times \frac{Precision\times Recall}{Precision+Recall}$$

(4) Average precision (AP)

(24) $$AP=\int_{0}^{1}Precision(Recall)\;\;d(Recall)$$

(5) Mean average precision (mAP)

(25) $$mAP=\frac{1}{n}\sum_{1}^{n}AP_{i}$$

#### 3.1.3. Experimental Results

**Figure 10:**
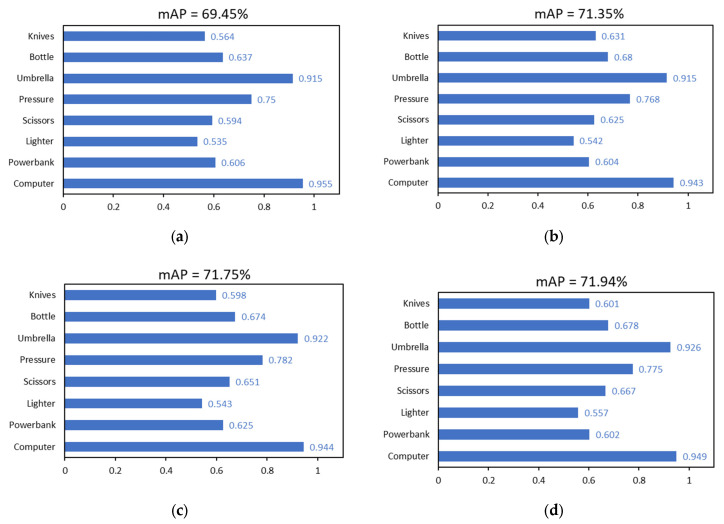
The comparative experiment of detection accuracy of YOLOv8s and other improved models on all kinds of contraband. (**a**) YOLOv8s, (**b**) YOLOv8s-EMA, (**c**) YOLOv8s-DCN, (**d**) YOLOv8s-DCN-EMA.

To verify the detection performance of the improved models based on deformable convolution (DCNv2) and channel attention (EMA), the YOLOv8s model with higher accuracy but more parameters and slower detection was compared with the YOLOv8n model with lower accuracy but fewer parameters and faster detection. According to the methods and principles introduced in Section 2.2 and Section 2.3, the deformable convolution model and the channel attention mechanism model were integrated and embedded into the above two models, and two models, YOLOv8s-DCN-EMA and YOLOv8n-DCN-EMA, were obtained, respectively. According to the configuration described in Section 3.1.1, 4800 images obtained from security X-ray scans were divided into training set, verification set and test set according to the ratio of 7:1.5:1.5. The data set contained eight types of items to be detected, including computer, power bank, lighter, scissors, pressure bottle, umbrella, water bottle and knife. The four models YOLOv8s-DCN-EMA, Yolov8n-DCN-EMA, YOLOv8s and YOLOv8n were trained with 3360 training set images. When the model was trained to the 100th round, the returned loss function had reached the convergence state. The detection and recognition effects of the original and revised model were then tested using 720 test set photos. The results of the experiments are displayed in the Table 1 and Figure 10. In general, it can be seen that in the test set, the mAP values corresponding to the best model trained by YOLOv8s, Yolov8s-DCN-EMA, YOLOv8n, Yolov8n-DCN-EMA are 69.45%, 71.94%, 62.65% and 66.06%, respectively. Compared to the initial model, the YOLOv8s-DCN-EMA model optimized by deformable convolution and channel attention has a detection effect mAP that is 2.49% greater. This suggests that the YOLOv8s-DCN-EMA has a stronger ability to extract features and discriminate between different types of information. The enhanced model has significantly increased the detection accuracy of multiple small contraband objects in each category of detection tasks, including lighters, scissors, water bottles, and knives. The corresponding improvement values are 2.2%, 7.3%, 4.1%, and 3.7%, respectively, which indicates that the improved module greatly affects the detection task of small targets. Similarly, the upgraded YOLOV8N-DCN-EMA model’s detection effect is better than the original YOLOv8n model’s detection effect, with an increase of 3.41%, and significant improvements have been made in the model’s overall detection performance and the detection performance of a single item. The YOLOv8s model series contains a comparatively larger amounts of parameters than the YOLOv8n model. This is because the model is better at extracting features, which leads to a higher detection accuracy.

Meanwhile, to verify the impact of a single improvement on the original model, this paper also conducted the experimental verification of four models, YOLOv8s-EMA, YOLOv8s-DCN, YOLOv8n-EMA and YOLOv8n-DCN. After testing, adding a single module will also improve the accuracy of the improved model detection. Taking the improved YOLOv8s model as an example, the overall detection accuracy mAP of the Yolov8s-EMA model is 71.35%, 1.90% higher than that of the YOLOv8s model. The general detection accuracy mAP of the YOLOv8s-DCN model is 71.75%, which is 2.30% higher than that of YOLOv8s model. The model with deformable convolution added has better recognition accuracy regarding the power bank, lighter, scissors, pressure bottle and umbrella than the model with the channel attention part added, while the model with the channel attention part added has better detection accuracy regarding the water bottle and the knife than the model with deformable convolution added. When the two methods are embedded in the initial model, the obtained YOLOv8s-DCN-EMA model generally improves the mAP of the whole and different kinds of items compared with the single improved method. The same is true for the enhanced YOLOv8n model. To sum up, although the two improvements have different enhancement effects on different categories of items, they will greatly improve the entire task of contraband detection, and the effect brought on by the combination of the two is greater than the original method. 

### 3.2. Test Experiment of Detection Model Based on Data Enhancement 

**Figure 11:**
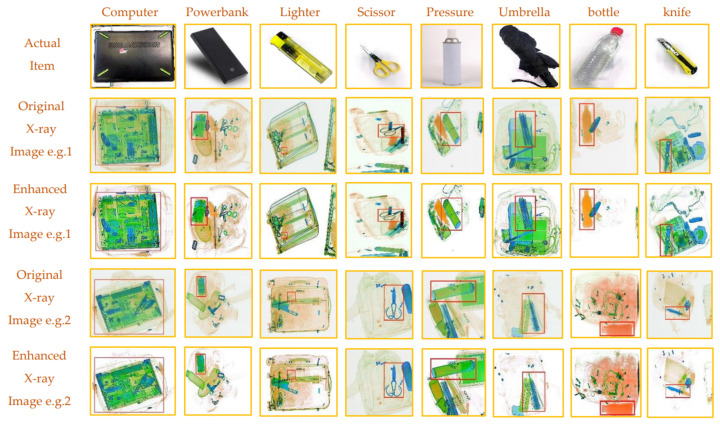
Samples of eight types of contraband and corresponding X-ray scan images and enhanced X-ray scan images.

The collected security luggage data set contains low-resolution photos, limiting the model’s capacity to extract features and resolve primary and secondary information. Consequently, in order to produce high-resolution images and enhance the model’s detection accuracy, super-resolution reconstruction experiments using the SRGAN model are required. Prior to the super-resolution reconstruction, brightness is added to the data. Next, we use a DIV dataset of 25,000 high-resolution images to train the generator and discriminator for the SRGAN super-resolution reconstruction model. The configuration used for training is the same as described in Section 3.1.1., i.e., training 100 rounds, having the batch size set to eight, and using the Adam optimizer training. The initial learning rate is set at 0.0002. According to the principle described in Section 2.1, in the process of training the discriminator, the program will randomly select a batch size of high-definition and real images, adjust the size to obtain low-resolution images, and then pass the batch size of high-definition images to the generator to generate. The label of the real HD image is set to 1, the generated HD image is set to 0, and then it is passed to the discriminator for training. Regarding the process of training the generator, it is mainly to pass the low-definition image into the generator to generate the high-definition image and output the score D through the discriminator to calculate the loss-logD to combat the loss. Then, the real HD image and the generated HD image are passed into the VGG network, respectively, to extract features, and the perceptual loss is calculated according to MSE. Finally, the total generator loss is calculated, and the network parameters of the generator are updated according to the backpropagation. The model’s ideal weight is determined following training and is maintained until the model converges. At this point, the generator’s ideal weight is loaded, and 4800 images from the original data set are used to perform super-resolution reconstruction, creating the super-resolution data set. Figure 11 shows the newly obtained images and the comparison between the old and new images. The contraband in the pictures have been marked with red borders.

**Figure 12:**
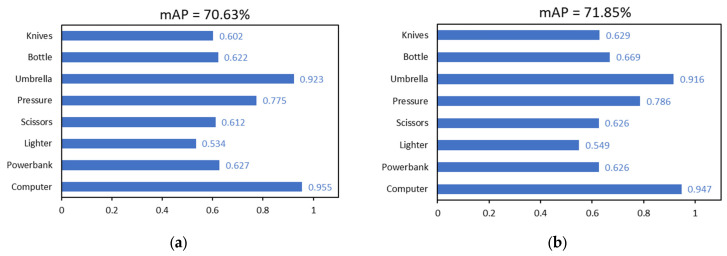

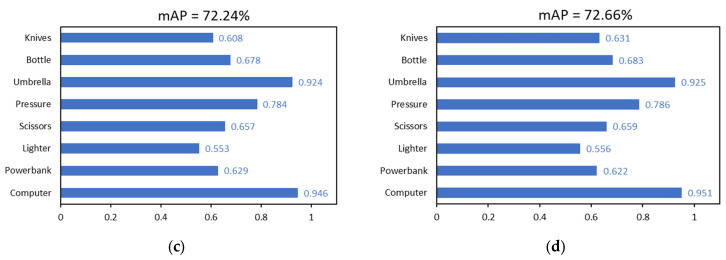
The comparative experiment of detection accuracy of YOLOv8s and other improved models based on SRGAN and data enhancement of all kinds of contraband. (**a**) YOLOv8s*, (**b**) YOLOv8s-EMA*, (**c**) YOLOv8s-DCN*, (**d**) YOLOv8s-DCN-EMA*.

To validate the impact of the data set on the precision of the model following data enhancement, the training set divided by the data set was utilized to train the YOLOv8 and YOLOv8-DCN-EMA models. The partitioning of the data sets was consistent with Section 3.1.1, with a ratio of 7:1.5:1.5. The setting of experimental parameters was also the same as in Section 3.1.1, and the image input size was 640 × 640. Since the improved model needs to be used in actual airport security checks, it is more appropriate to use the original test data as the test set, which can better reflect the model’s capacity for generalization. The results of the experiments are displayed in the Table 2 and Figure 12. YOLOv8s*, Yolov8S-DCN-EMA*, YOLOv8n*, and Yolov8N-DCN-EMA* represent the four improved models. The models obtained after image enhancement training are marked with * in the upper right corner shown below. It can be found that no matter the YOLOv8n series and its improved model or the YOLOv8s series and its improved model, the detection effect has been greatly improved. Taking the YOLOv8 series as an example, the detection effect of YOLOv8s-DCN-EMA* was the best, and its mAP value was 72.96%. 

Compared with other groups, the detection effect of YOLOv8s-DCN-EMA* was 1.02% worse than that of YOLOv8s-DCN-EMA*. The detection effect mAP of YOLOv8s was 1.18% worse than that of YOLOv8s*. Similarly, in this group of experiments, we also conducted experiments on the model with a single module added as a comparison, and we can see that the detection effect of the model trained on the dataset after super-resolution reconstruction was better than that of the dataset without super-resolution reconstruction. For example, the detection effect mAP of YOLOv8s-DCN* was 0.49% higher than that of YOLOv8s-DCN. The detection effect mAP of YOLOv8s-EMA* was 0.50% higher than that of YOLOv8s-EMA. For the detection effect of different categories of items, the training model on the improved data set had a better detection effect than the training model before the improvement. Thus, this experiment demonstrates that when training the security contraband detection model, the more varied the data set content, the higher the image quality, the more robust the model’s ability of feature extraction and resolve information, and the more effective the corresponding detection effect.

### 3.3. Detection Model Test Experiment Based on an Improved Pigeon-Inspired Algorithm to Optimize Model Learning Rate

**Figure 13:**
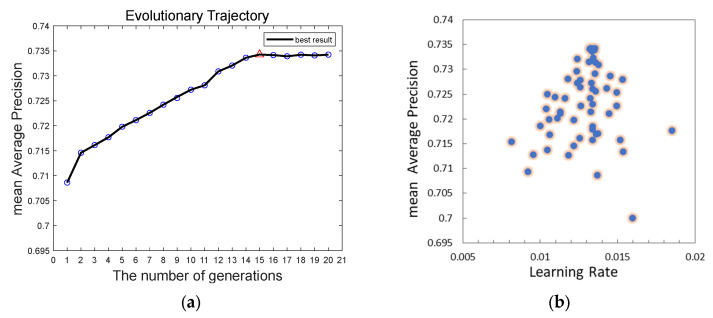
Evolution and optimization of improved pigeon-inspired optimization. (**a**) Outer-cycle evolution curve, (**b**) Optimization process.

**Figure 14:**
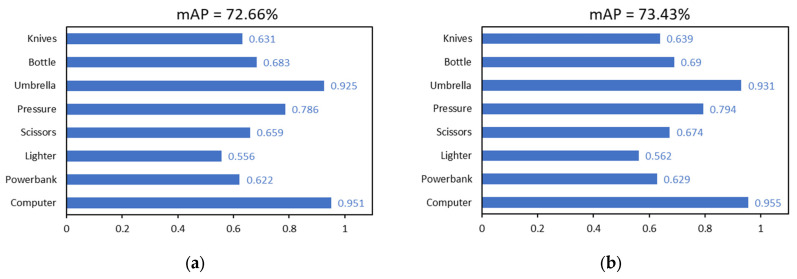
The YOLOv8s-DCN-EMA-IPIO* detection model and the YOLOv8s-DCN-EMA* detection model are compared experimentally in this diagram. (**a**) YOLOv8s-DCN-EMA*, (**b**) YOLOv8s-DCN-EMA-IPIO*.

We provide an enhanced pigeon-inspired optimization technique based on the cross-mutation strategy to optimize the learning rate parameters in the hyperparameters and raise the detection accuracy by mimicking the homing properties of pigeons. The improved flock method states that four pigeons N are in the flock and that each pigeon represents the learning rate. The learning rate has three limits: 0.1 for the upper limit, 0.001 for the lower limit, and 0.3 for the compass factor. The compass operator’s maximum number of iterations is 12, the landmark operator’s maximum number of iterations is 8, and the maximum number of iterations is 20. The corresponding parameters are updated with each iteration round. The model input of YOLOv8s-DCN-EMA* is 640 × 640, the batch size is eight, and the training times are 100 rounds according to the description in Section 3.2. The process of training the model is called internal cycle training here. In the iteration process of the compass operator, since the number of pigeons is set to four, each iteration optimization includes four inner-loop training. Each parameter update iteration operation is called the outer-loop evolution. The detection accuracy mAP value obtained from each inner-cycle training is used as the fitness value of the flock, while the highest accuracy value obtained from each outer-cycle evolution is called the optimal fitness value, that is, the optimal mAP. At this time, the next generation’s flock position is updated according to the learning rate corresponding to the optimal mAP and the compass operator’s updated formula, which is the learning rate required for the next round of outer-loop evolutionary training. The fitness value obtained via the next generation of outer-loop evolution which is sorted to obtain the corresponding optimal fitness value and so on until the number of iterations reaches the maximum number of iterations of the compass operator. At this time, the compass operator is stopped, and the landmark operator is used. Instead, the number of pigeons is halved by the generation, and the optimal mAP value is obtained through inner-cycle training and precision value order. We then perform iterative training based on the improved pigeon-inspired optimization algorithm of the YOLOv8s-DCN-EMA* model using a training set and a validation set based on data enhancement. As in Section 3.2, untrained raw data sets are used as test sets to demonstrate the generalization ability of the model. The final experimental results are displayed in Figure 13 and Figure 14 and Table 3, and the experimental setup and conditions are the same. In the test of the model obtained via the 15th round of external cyclic evolution, the optimal mAP is obtained, which is 73.43%, and the corresponding optimal learning rate is 0.01354.

The results of the experiments reveal that the mAP of the YOLOv8s-DCN-EMA* model with an optimized learning rate is 0.77% better than that of the non-optimized model, and the overall detection performance is improved. Meanwhile, the detection accuracy of each prohibited item has been improved accordingly. In the operation of outer-loop evolution, when the number of iteration training reaches the 15th round, the optimal mAP measured by the model on the test set reaches the highest, and the mAP value in the next five rounds gradually becomes stable and no longer rises. It can be seen from Figure 13b that the pigeons will eventually reach the destination. That is, the points corresponding to the optimal learning rate and the optimal mAP will converge generation by generation and eventually converge to one location. In conclusion, the cross-mutation strategy-based modified pigeon swarm algorithm may dynamically optimize the model’s learning rate, allowing it to converge to the ideal learning rate more quickly and effectively while enhancing detection performance and accuracy.

### 3.4. Ablation Study and Analysis

To verify the validity of all modules in the YOLOv8s-DCN-EMA-IPIO* model, the following ablation experiments were performed with the same experimental parameters and configurations. As can be seen from the Table 4, adding one or two modules will eventually improve the overall detection accuracy value mAP. After adding the DCN module alone, the overall detection accuracy of the model is the best, at 2.3%. Adding the EMA attention module alone and the addition of data enhancement processing resulted in increased changes of 1.1% and 1.9%, respectively, indicating that the DCN module was able to focus better and pay attention to the size and shape characteristics of objects. In terms of the accuracy rate, recall rate and F1 parameters, it is noted that models with data enhancement and SRGAN super-resolution reconstruction modules have a noticeable improvement in the recall rate, while the improvement effect brought on by the DCN module and the EMA module is mainly reflected in the accuracy rate. The mAP (50–95) corresponds to mAP and is an improvement over the original model. In the meantime, the improvement effect brought on by the introduction of SRGAN super-resolution reconstruction, the DCN module, and the EMA module has been greatly improved in the above five detection and evaluation indicators. Adding the improved learning rate of pigeon-inspired optimization based on the former can increase the mAP by 0.77%, and other indicators are also improved. Finally, in terms of FPS parameters, it was found that the calculation rate of the five models would be reduced somewhat with the introduction of the DCN module. However, the difference was not large compared with the original, because the addition of a deformable convolutional module would lead to more model parameters, a larger calculation quantity and a longer inference time. At the same time, introducing the EMA attention module also resulted in a slower speed and a lower amplitude. In summary, the improved model improves the accuracy and does not affect the detection speed too much and can still be used in practical applications.

### 3.5. Comparative Experiment of Performance of Different Models

To confirm the effectiveness of the model even more, this paper compares the YOLOv8s-DCN-EMA and YOLOv8s-DCN-EMA-IPIO* models with the current mainstream general target detection algorithms, and the results are shown in Table 5. The algorithms involved in the comparison include Faster RCNN [27], DETR, RT-DETR-L, and YOLO series detection models. Compared with the detection algorithm Faster RCNN, YOLOv8s-DCN-EMA improved by 7.1% in the mAP index and 5.6% in the mAP (50–95), and FPS also improved correspondingly. Compared with DETR, the object detection algorithm of the past two years, and RT-DETR-L, the latest real-time object detection algorithm, YOLOv8s-DCN-EMA increased the mAP index by 1.8% and 0.6%, respectively, while the number of parameters and the calculation amount decreased significantly. The parameters of YOLOv8s-DCN-EMA were 27.7% and 35.0% of those of DETR and RT-DETR-L [53,54], respectively. In addition, FPS also increased significantly. Meanwhile, the detection algorithm of the YOLO series, YOLOv8s-DCN-EMA and YOLOv3-tiny [42], YOLOv5s, YOLOv6s-ReLU [55], YOLOv7-tiny [44], YOLOv8n, YOLOv8s, and other lightweight networks are compared. It can be concluded that the mAP index increased by 8.4%, 4.1%, 5.8%, 4.9%, 9.2%, and 2.4%, respectively. In the mAP (50–95), it increased by 7.8%, 2.2%, 3.1%, 2.3%, 7.4%, and 1.6%, respectively. Compared with the above models, the change in the parameter number and calculation amount is not obvious. It can be shown that compared with other mainstream target detection algorithms, YOLOv8s-DCN-EMA and YOLOv8s-DCN-EMA-IPIO* algorithms achieve an extremely high detection accuracy without too much impact on the detection speed, under the premise of reducing the number of parameters and the calculation amount, and strike a balance between being lightweight and having accuracy. It fits the characteristics of lightweight, high precision and high efficiency in the public security system, and is suitable for deployment on low-cost equipment with limited computing resources.

### 3.6. The Comparison Results with Related Strategies

In order to verify the effectiveness and superiority of the added attentional mechanisms, we added CA, ECA, CBAM, SE, and EMA to the YOLOv8s model, respectively, to test the effect of the improved model. The experimental results are shown in Table 6. From Table 6, it can be seen that the EMA attention module greatly improves the detection accuracy of the network compared to other attention modules, such as ECA, CBAM.

To verify the performance improvement effect of optimizing the deep learning hyperparameters under different configurations, we added IPIO to YOLOv8s, YOLOv8s-DCN, YOLOv8s-EMA, YOLOv8s-DCN-EMA, and YOLOv8s-DCN-EMA* to test the optimized effect, respectively. The experimental results are shown in Table 7. Optimizing the learning rate in different models can all improve the final detection accuracy. The highest final detection accuracy value of 73.4% was obtained by introducing the IPIO algorithm to optimize the learning rate in the YOLOv8s-DCN-EMA* model.

### 3.7. Validation of the Generalization Ability of the Model

To test the generalization ability of the model, this paper places the model on different data sets for experiments. We collected 1200 images from the airport and filtered and calibrated these images to finally retrieve 700 images as the test set, which contained eight types of contraband: computers, rechargeable batteries, lighters, scissors, compressed bottles, umbrellas, water bottles, and pocket knives. The YOLOv8s model and the YOLOv8s-DCN-EMA-IPIO* model were run on the data set and the following results were obtained, which are shown in Table 8. As can be seen from Table 8, the mAP and mAP (50–95) of the YOLOv8s-DCN-EMA-IPIO* model are 72.8% and 50.1%, respectively. Compared with the above, there is a slight decrease in the detection accuracy value, but the decrease is not significant and is within 1%, indicating that the model has some generalization ability. Meanwhile, the FPS is 95, so it can also meet the demand of real-time performance and accuracy in practical applications.

## 4. Conclusions

Taking the detection and identification of security contraband as the research target, this paper proposes a network structure based on YOLOv8s, aiming at solving the problems of missing and false detection of X-ray contraband in actual situations. YOLOv8s-DCN-EMA-IPIO* combines data enhancement, the deformable convolutional DCNv2, the multi-scale attention mechanism EMA, and the automatic hyperparameter optimization model. The deformable convolutional DCNv2 is used to replace the C2f module in the backbone network and make it become the C2f_DCN layer to increase the sensitivity field of contraband of small sizes and different shapes, thus improving the detection performance of the model on small and medium target objects. The neck network includes the multi-scale attention mechanism known as EMA to reduce interference from complicated background noise and overlapping occlusion phenomena during the detection phase. This mechanism forces the model to focus more on primary information and disregard secondary information. Data enhancement and SRGAN super-resolution reconstruction technology transform low-resolution X-ray security images into super-resolution images. This process improves the quality of model training data sets and increases the enhanced model’s detection accuracy. Convolutional neural networks use an improved pigeon-inspired optimization based on the cross-mutation method to optimize the hyperparameters, precisely the learning rate. The location of the flock is constantly updated throughout the global search and iteration process. It not only realizes the optimal selection of the initial position of the flock, but also obtains the optimal position of the pigeons through fast convergence, that is, the optimal learning rate, and then receives better detection and recognition accuracy of the model. Through experimental inspection, the mAP of the improved model YOLOV8s-DCN-EMA-IPIO* is 3.98% better than that of the model YOLOv8s, and the accuracy and recall rate are also improved, which are 6.8% and 2.1%, respectively. Due to the large amount of computation of the algorithm, the next step is to reduce the number of parameters and the amount of computation through a lightweight network while ensuring the same detection accuracy to improve further the detection speed and real-time performance of the model, which is convenient for embedded landing and development.

## Figures and Tables

**Table 1:** Comparative experiment of detection accuracy of different improved models on all kinds of contraband. Among them, YOLOv8s is abbreviated as Y8s, yolov8s-DCN is abbreviated as Y8sD, yolov8s-EMA is abbreviated as Y8sE, yolov8s-DCN-EMA is abbreviated as Y8sDE, and the rest are the same in the following table.

Model	mAP/%	Categories (AP)/%							
		Computer	Powerbank	Lighter	Scissors	Pressure	Umbrella	Bottle	Knives
Y8n	62.65	94.0	51.2	41.0	45.8	68.2	87.5	63.1	50.4
Y8nE	64.95	94.2	53.9	43.6	48.7	70.9	89.8	65.7	52.8
Y8nD	65.55	94.9	52.1	41.2	52.9	73.1	89.5	62.6	58.1
Y8nDE	66.06	94.7	52.8	41.4	53.9	69.8	90.5	65.8	59.6
Y8s	69.45	95.5	60.6	53.5	59.4	75.0	91.5	63.7	56.4
Y8sE	71.35	94.3	60.4	54.2	62.5	76.8	91.5	68.0	63.1
Y8sD	71.75	94.4	62.5	54.3	65.1	78.2	92.2	67.5	59.8
Y8sDE	71.94	94.9	60.2	55.7	66.7	77.5	92.6	67.8	60.1

**Table 2:** Comparative experiment of detection accuracy of different improved models based on SRGAN and data enhancement of all kinds of contraband.

Model	mAP/%	Categories (AP)/%							
		Computer	Powerbank	Lighter	Scissors	Pressure	Umbrella	Bottle	Knives
Y8n*	64.68	94.5	53.5	42.8	48.1	70.5	89.6	65.9	52.5
Y8nE*	66.13	94.7	53.7	43.8	51.6	72.3	89.4	65.7	57.8
Y8nD*	66.85	95.2	53.9	44.3	53.7	73.5	89.6	66.1	58.5
Y8nDE*	67.48	95.3	54.3	44.8	54.5	73.9	90.8	66.2	60.0
Y8s*	70.63	95.5	62.7	53.4	61.2	77.5	92.3	62.2	60.2
Y8sE*	71.85	94.7	62.6	54.9	62.6	78.6	91.6	66.9	62.9
Y8sD*	72.24	94.6	62.9	55.3	65.7	78.4	92.4	67.8	60.8
Y8sDE*	72.66	95.1	62.2	55.6	65.9	78.6	92.5	68.3	63.1

**Table 3:** The experimental comparison between the YOLOv8s-DCN-EMA-IPIO* detection model and the YOLOv8s-DCN-EMA* detection model.

Model	mAP/%	Categories (AP)/%							
		Computer	Powerbank	Lighter	Scissors	Pressure	Umbrella	Bottle	Knives
Y8sDE*	72.66	95.1	62.2	55.6	65.9	78.6	92.5	68.3	63.1
Y8sDEP*	**73.43**	**95.5**	**62.9**	**56.2**	**67.4**	**79.4**	**93.1**	**69.0**	**63.9**

The bolded font indicates the experimental results of the improved final model in this article.

**Table 4:** The results of the ablation experiment.

YOLOv8s	SRGAN	DCN	EMA	IPIO	Precision	Recall	F1 Score	mAP	mAP (50–95)	FPS
✓					71.4%	65.2%	68.2%	69.5%	47.4%	123
✓	✓				73.2%	65.8%	69.3%	70.6%	47.9%	124
✓		✓			75.6%	64.7%	69.7%	71.8%	48.9%	96
✓			✓		74.1%	64.4%	68.9%	71.4%	48.8%	111
✓	✓	✓			76.4%	64.8%	70.1%	72.2%	49.5%	96
✓	✓		✓		74.8%	66.5%	70.4%	71.9%	49.3%	112
✓		✓	✓		75.3%	64.9%	69.9%	71.9%	49.0%	91
✓	✓	✓	✓		77.7%	66.2%	71.5%	72.7%	50.3%	92
**✓**	**✓**	**✓**	**✓**	**✓**	**78.2%**	**67.3%**	**72.3%**	**73.4%**	**50.6%**	**95**

The bolded font indicates the experimental results of the improved final model in this article.

**Table 5:** Comparative experiments with different detection models.

Model	mAP/%	mAP (50–95)/%	Params/M	FLOPs/G	FPS (GPU)
Faster RCNN	64.8	43.4	137.1	370.3	54
DETR	70.1	44.2	41.5	100.9	56
RT-DETR-L	71.3	46.5	32.9	110.2	78
YOLOv3-tiny	63.5	41.2	12.1	19.2	238
YOLOv5s	67.8	46.8	7.1	16.7	167
YOLOv6s-ReLU	66.1	45.9	16.3	42.8	152
YOLOv7-tiny	67.0	46.7	6.0	13.2	189
YOLOv8n	62.7	41.6	3.1	8.2	255
YOLOv8s	69.5	47.4	10.9	28.4	123
YOLOv8s-DCN-EMA	71.9	49.0	11.5	30.6	91
YOLOv8s-DCN-EMA-IPIO*	**73.4**	**50.6**	**11.5**	**30.6**	**95**

The bolded font indicates the experimental results of the improved final model in this article.

**Table 6:** Comparison of networks with various attention mechanism modules.

Model	mAP/%	mAP (50–95)/%	Params/M	FLOPs/G	FPS(GPU)
YOLOv8s	69.5	47.4	10.9	28.4	123
YOLOv8s + CA	70.7	48.3	11.0	28.6	116
YOLOv8s + ECA	71.1	48.4	10.9	28.5	114
YOLOv8s + CBAM	70.9	48.1	11.1	28.6	112
YOLOv8s + SE	70.3	47.9	11.0	28.5	119
**YOLOv8s + EMA**	**71.4**	**48.8**	**11.2**	**28.9**	**111**

The bolded font indicates the experimental results of the YOLOv8s model with EMA in this article.

**Table 7:** Comparative experiments to optimize model learning rate under different configurations.

Model	mAP/%	mAP (50–95)/%	Params/M	FLOPs/G	FPS(GPU)
YOLOv8s	69.5	47.4	10.9	28.4	123
YOLOv8s **+ IPIO**	**70.4**	**48.0**	**10.9**	**28.4**	**125**
YOLOv8s + EMA	71.4	48.8	11.2	28.9	111
YOLOv8s + EMA **+ IPIO**	**72.3**	**49.3**	**11.2**	**28.9**	**112**
YOLOv8s + DCN	71.8	48.9	11.4	29.5	96
YOLOv8s + DCN **+ IPIO**	**72.6**	**49.4**	**11.4**	**29.5**	**98**
YOLOv8s + DCN + EMA	71.9	49.0	11.5	30.6	91
YOLOv8s + DCN + EMA **+ IPIO**	**72.8**	**49.7**	**11.5**	**30.6**	**93**
YOLOv8s + DCN + EMA*	72.7	50.3	11.5	30.6	92
YOLOv8s + DCN + EMA* **+ IPIO**	**73.4**	**50.6**	**11.5**	**30.6**	**95**

The bolded font indicates the experimental results of the improved YOLOv8s model with IPIO in this article.

**Table 8:** Comparative experiments to validate the generalization ability of models.

Model	mAP/%	mAP (50–95)/%	Params/M	FLOPs/G	FPS (GPU)
YOLOv8s	68.7	46.2	10.9	28.4	123
YOLOv8s-DCN-EMA-IPIO*	**72.8**	**50.1**	**11.5**	**30.6**	**95**

The bolded font indicates the experimental results of the improved final model in this article.

## Data Availability

Data are not available due to privacy restrictions.

## References

[1] European Parliament (2012). Aviation Security with a Special Focus on Security Scanners. European Parliament Resolution of 6 July 2011 on Aviation Security, with a Special Focus on Security Scanners (2010/2154(INI)). https://eur-lex.europa.eu/legal-content/EN/TXT/PDF/?uri=CELEX:52011IP0329&rid=1.

[2] Mery D., Svec E., Arias M., Riffo V., Saavedra J.M., Banerjee S. (2017). Modern Computer Vision Techniques for X-Ray Testing in Baggage Inspection. IEEE Trans. Syst. Man Cybern. Syst..

[3] Schwaninger A., Bolfing A., Halbherr T., Helman S., Belyavin A., Hay L. The impact of image based factors and training on threat detection performance in X-ray screening. Proceedings of the 3rd International Conference on Research in Air Transportation, ICRAT 2008.

[4] Blalock G., Kadiyali V., Simon D.H. (2007). The impact of post-9/11 airport security measures on the demand for air travel. J. LawEcon..

[5] Hou Y. (2018). Research on the Relationship between Work-Stress and Safety Performance of Airport Security Inspectors. Master’s Thesis.

[6] Michel S., Koller S.M., de Ruiter J.C., Moerland R., Hogervorst M., Schwaninger A. Computer-based training increases efficiency in X-ray image interpretation by aviation security screeners. Proceedings of the 2007 41st Annual IEEE International Carnahan Conference on Security Technology.

[7] Wu Y., Zhao X., Jin Y., Zhang X. (2013). Application of edge detection operator in extracting golden region of image. Beijing Inst. Print. Technol. J..

[8] Mei H. (2015). Research and Application of Contour Extraction Method for Moving Objects in Surveillance Video. Master’s Thesis.

[9] Su B., Chen J., Chen Y. (2019). X-ray Image Contraband Classification Method Based on Joint Feature. Digit. Technol. Appl..

[10] Wang Y., Zhou W.H., Yang X.M., Jiang W., Wu W. (2017). Classification of foreign bodies in X-ray images based on computer vision. Chin. J. Liq. Cryst. Disp..

[11] Han P., Liu Z., He W. (2011). An effective two-stage enhancement method for Airport Security X-ray carry-on image. Photoelectronics.

[12] Krizhevsky A., Sutskever I., Hinton G.E. ImageNet Classification with Deep Convolutional Neural Networks. Proceedings of the Advances in Neural Information Processing Systems.

[13] Szegedy C., Ioffe S., Vanhoucke V., Alemi A.A. Inception-v4, inception-ResNet and the impact of residual connections on learning. Proceedings of the AAAI Conference on Artificial Intelligence.

[14] Zhu Y., Newsam S. DenseNet for dense flow. Proceedings of the 2017 IEEE International Conference on Image Processing (ICIP).

[15] Bastan M., Yousefifi M.R., Thomas M.B. (2011). Visual words on baggage X-ray images. International Conference on Computer Analysis of Images and Patterns.

[16] Jongseo P., Minjoo C. (2022). A k-means Clustering Algorithm to Determine Representative Operational Profiles of a Ship Using AIS Data. J. Mar. Sci. Eng..

[17] Esteve M., Aparicio J., Rodriguez-Sala J.J., Zhu J. (2023). Random Forests and the measurement of super efficiency in the context of Free Disposal Hull. Eur. J. Oper. Res..

[18] Hearst M.A., Dumais S.T., Osuna E., Platt J., Scholkopf B. (1998). Support vector machines. IEEE Intell. Syst. Appl..

[19] Mery D., Svec E., Arias M. (2016). Object recognition in baggage inspection using adaptive sparse representations of X-ray images. Proceedings of the PSIVT 2015: Image and Video Technology.

[20] Mery D., Riffo V., Zuccar I., Pieringer C. Automated X-ray object recognition using an efficient search algorithm in multiple views. Proceedings of the 2013 IEEE Conference on Computer Vision and Pattern Recognition Workshops.

[21] Mery D., Mondragon G., Riffo V., Zuccar I. (2013). Detection of regular objects in baggage using multiple X-ray views. Insight-Non-Destr. Test. Cond. Monit..

[22] Wu H.-B., Wei X.-Y., Liu M.-H., Wang A.-L., Liu H., Iwahori Y. (2021). Improved YOLOv4 for dangerous goods detection in X-ray inspection combined with atrous convolution and transfer learning. Chin. Opt..

[23] Dong Y., Li Z., Guo J., Chen T., Lu S. (2023). An improved YOLOv5 model for X-ray prohibited items detection. Laster Optoelectron. Prog..

[24] Liu W., Anguelov D., Erhan D., Szegedy C., Reed S., Fu C.-Y., Berg A.C. (2016). SSD: Single shot multibox detector. European Conference on Computer Vision.

[25] Zhang Y.K., Su Z.G., Zhang H.G., Yang J.F. (2020). Multi-scale Prohibited Item Detection in X-ray Security Image. J. Signal Process..

[26] Guo S., Zhang L. (2021). Yolo-C: One-stage network for prohibited items detection within X-ray images. Laser Optoelectron. Prog..

[27] Ren S., He K., Girshick R., Sun J. Faster R-CNN: Towards real-time object detection with region proposal networks. Proceedings of the Advances in Neural Information Processing Systems.

[28] Lin T.-Y., Dollár P., Girshick R., He K., Hariharan B., Belongie S. Feature pyramid networks for object detection. Proceedings of the IEEE Conference on Computer Vision and Pattern Recognition.

[29] Gao R., Sun Z., Huyan J., Li W., Xiao L., Yao B., Wang H. (2021). Small Foreign Metal Objects Detection in X-Ray Images of Clothing Products Using Faster R-CNN and Feature Pyramid Network. IEEE Trans. Instrum. Meas..

[30] Wei Y., Tao R., Wu Z., Ma Y., Zhang L., Liu X. Occluded prohibited items detection: An X-ray security inspection benchmark and de-occlusion attention module. Proceedings of the 28th ACM International Conference on Multimedia.

[31] Zhang N., Luo Y., Bao X., Jin Y., Tu X. (2022). X-ray Security Inspection for Contraband Detection Based on Improved Cascade RCNN Network. Comput. Syst. Appl..

[32] You X., Hou J., Ren D., Yang P., Du M. (2023). Adaptive Security Check Prohibited Items Detection Method with Fused Spatial Attention. Comput. Eng. Appl..

[33] Wang Z., Xu H., Zhu X., Li S., Liu Z., Wang Z. (2023). Improved Dense pedestrian detection algorithm based on YOLOv8: MER-YOLO. Comput. Eng. Sci..

[34] Gao A., Liang X., Xia C., Zhang C. (2023). An Improved YOLOv8 Dense pedestrian detection algorithm. J. Graph..

[35] Li S., Shi T., Jing F. (2023). Improved Road damage detection algorithm of YOLOv8. Comput. Eng. Appl..

[36] Leng R. (2023). Application of Foreign Objects Identification of Transmission Lines Based on YOLOv8 Algorithm. Master’s Thesis.

[37] Goodfellow I., Pouget-Abadie J., Mirza M., Xu B., Warde-Farley D., Ozair S., Courville A., Bengio Y. Generative adversarial networks. Proceedings of the Advances in Neural Information Processing Systems.

[38] Ledig C., Theis L., Huszar F., Caballero J., Cunningham A., Acosta A., Aitken A., Tejani A., Totz J., Wang Z. Photo-realistic single image super-resolution using a generative adversarial network. Proceedings of the IEEE Conference on Computer Vision and Pattern Recognition.

[39] He K., Zhang X., Ren S., Sun J. Deep residual learning for image recognition. Proceedings of the IEEE Conference on Computer Vision and Pattern Recognition.

[40] Redmon J., Divvala S., Girshick R., Farhadi A. You Only Look Once: Unified, Real-Time Object Detection. Proceedings of the IEEE Conference on Computer Vision and Pattern Recognition.

[41] Redmon J., Farhadi A. YOLO9000: Better, Faster, Stronger. Proceedings of the IEEE Conference on Computer Vision and Pattern Recognition.

[42] Redmon J., Farhadi A. (2018). YOLOv3: An Incremental Improvement. arXiv.

[43] Bochkovskiy A., Wang C.Y., Liao H.Y.M. (2020). YOLOv4: Optimal Speed and Accuracy of Object Detect-ion. arXiv.

[44] Wang C.Y., Bochkovskiy A., Liao H.Y.M. YOLOv7: Trainable bag-of-freebies sets new state-of-the-art for real-time object detectors. Proceedings of the IEEE/CVF Conference on Computer Vision and Pattern Recognition.

[45] Ouyang D., He S., Zhang G., Luo M. Efficient Multi-Scale Attention Module with Cross-Spatial Learning. Proceedings of the IEEE International Conference on Acoustics, Speech and Signal Processing (ICASSP).

[46] Woo S., Park J., Lee J.Y., Kweon I.S. CBAM: Convolutional block attention module. Proceedings of the European Conference on Computer Vision (ECCV).

[47] Hu J., Shen L., Sun G. Squeeze-and-Excitation Networks. Proceedings of the IEEE Conference on Computer Vision and Pattern Recognition.

[48] Hou Q., Zhou D., Feng J. Coordinate Attention for Efficient Mobile Network Design. Proceedings of the IEEE Conference on Computer Vision and Pattern Recognition.

[49] Wang Q., Wu B., Zhu P., Li P., Zuo W., Hu Q. ECA-Net: Efficient Channel Attention for Deep Convolutional Neural Networks. Proceedings of the IEEE Conference on Computer Vision and Pattern Recognition.

[50] Dai J., Qi H., Xiong Y., Li Y., Zhang G., Hu H., Wei Y. Deformable convolutional networks. Proceedings of the IEEE International Conference on Computer Vision.

[51] Zhu X., Hu H., Lin S., Dai J. Deformable convnets v2:more deformable, better results. Proceedings of the IEEE/CVF Conference on Computer Vision and Pattern Recognition.

[52] Duan H., Qiao P. (2014). Pigeon-inspired optimization: A new swarm intelligence optimizer for air robot path planning. Int. J. Intell. Comput. Cybern..

[53] Carion N., Massa F., Synnaeve G., Usunier N., Kirillov A., Zagoruyko S. End-to-end object detection with transformers. Proceedings of the ECCV 2020.

[54] Lv W., Zhao Y., Xu S. (2023). DETRs Beat YOLOs on Real-time Object Detection. arXiv.

[55] Li C., Li L., Jiang H., Weng K., Geng Y., Li L., Ke Z., Li Q., Cheng M., Nie W. (2022). YOLOv6: A Single-Stage Object Detection Framework for Industrial Applications. arXiv.

